# Personalized approach in brain protection by hypothermia: individual changes in non-pathological and ischemia-related glutamate transport in brain nerve terminals

**DOI:** 10.1186/s13167-016-0075-1

**Published:** 2016-12-15

**Authors:** Artem Pastukhov, Natalia Krisanova, Vitalii Maksymenko, Tatiana Borisova

**Affiliations:** 1 The Department of Neurochemistry, Palladin Institute of Biochemistry, NAS of Ukraine, 9 Leontovicha Str, Kyiv, 01601 Ukraine; 2 Amosov Institute of Cardiovascular Surgery of the Academy of Medical Sciences of Ukraine, 6 N. Amosov Str, Kyiv, 03110 Ukraine; 3 Faculty of Biomedical Engineering, National Technical University of Ukraine “KPI”, 16/2 Yangel Str, Kyiv, 56 Ukraine

**Keywords:** Glutamate, Uptake and tonic release, Permanent glutamate turnover, Glutamate transporter reversal, Deep and profound hypothermia, Neuromonitoring, Individual hypothermia regime, Brain nerve terminals

## Abstract

**Background:**

Both deep and profound hypothermia are effectively applied in cardiac surgery of the aortic arch, when the reduction of cerebral circulation facilitates operations, and for the prevention of ischemic stroke consequences. Neurochemical discrimination of the effects of deep and profound hypothermia (27 and 17 °C, respectively) on non-pathological and pathological ischemia-related mechanisms of presynaptic glutamate transport with its potential contribution to predictive, preventive and personalized medicine (PPPM) was performed.

**Methods:**

Experiments were conducted using nerve terminals isolated from rat cortex (synaptosomes). Glutamate transport in synaptosomes was analyzed using radiolabel l-[^14^C]glutamate. Diameter of synaptosomes was assessed by dynamic light scattering.

**Results:**

Synaptosomal transporter-mediated uptake and tonic release of l-[^14^C]glutamate (oppositely directed processes, dynamic balance of which determines the physiological extracellular level of the neurotransmitter) decreased in a different range in deep/profound hypothermia. As a result, hypothermia-induced changes in extracellular l-[^14^C]glutamate are not evident (in one half of animals it increased, and in other it decreased). A progressive decrease from deep to profound hypothermia was shown for pathological mechanisms of presynaptic glutamate transport, that is, transporter-mediated l-[^14^C]glutamate release (*) stimulated by depolarization of the plasma membrane and (**) during dissipation of the proton gradient of synaptic vesicles by the protonophore FCCP.

**Conclusions:**

Therefore, the direction of hypothermia-induced changes in extracellular glutamate is unpredictable in “healthy” nerve terminals and depends on hypothermia sensitivity of uptake vs. tonic release. In affected nerve terminals (e.g., in brain regions suffering from a reduction of blood circulation during cardiac surgery, and core and penumbra zones of the insult), pathological transporter-mediated glutamate release from nerve terminals decreases with progressive significance from deep to profound hypothermia, thereby underlying its potent neuroprotective action. So, alterations in extracellular glutamate during hypothermia can be unique for each patient. An extent of a decrease in pathological glutamate transporter reversal depends on the size of damaged brain zone in each incident. Therefore, test parameters and clinical criteria of neuromonitoring for the evaluation of individual hypothermia-induced effects should be developed and delivered in practice in PPPM.

## Background

Hypothermia is effectively practiced in cardiac surgery to facilitate operations on the aortic arch with the reduction of cerebral circulation. The optimal degree of hypothermia for circulatory arrest in aortic arch surgery has been intensively debated, and the concept of using hypothermia to diminish the oxygen consumption and metabolic requirements of hypoxic tissues is rather intuitive [1]. The brain represents only 2–3% of human body weight; nevertheless, it uses 20–25% of the total consumption of oxygen and glucose [2]. Postoperative neurologic morbidity and mortality stay elevated and concerns over neurocognitive deficits caused by hypothermic neuronal injury have been raised [1, 3], despite data of the literature on complete neurocognitive preservation following deep hypothermic circulatory arrest [4]. Multiple studies using animal models that have attempted to clarify the optimal temperature for hypothermic circulatory arrest have generally supported the use of deeper levels of hypothermia; nevertheless, influence of a range of temperatures on the brain functioning needs to be better assessed [1]. In general, the mechanisms of the neuroprotective action of low temperatures during cardio surgery are a topical issue for medical practitioners.

Ischemic stroke leads to brain impairment and still remains one of the main causes of adult’s disability in developed countries. In the central core region of the insult, the neuronal cells are dead within minutes, whereas in the penumbral zone, the survival of the cells is probably achievable. Precise monitoring of brain temperature after stroke, traumatic brain injury, and subarachnoid hemorrhage is important because the brain is particularly susceptible and vulnerable to even small variations in the temperature [5–7]. Therapeutic hypothermia has long been known to be a nonspecific and potent neuroprotectant. It was observed in animal models that hypothermia reduced the size of cerebral infarcts by more than a half and this fact was a base for following clinical trials of therapeutic hypothermia in patients with ischemic stroke [7, 8]. In human stroke, administration of therapeutic hypothermia can improve short-term survival and neurological recovery and result in less extensive damage of the brain as it can be seen on computed tomography scans [9]. Experimental confirmation and clinical experience show that therapeutic hypothermia has a bright prospect for the administration in acute ischemic brain injury, and the profit is greatest when the treatment is started earlier, namely, within several hours of symptom onset. Low body temperature should be maintained for a longer period after stroke to reach long-lasting neuroprotective outcome [9–12]. Although until now, there are no comprehensible standard parameters of therapeutic hypothermia [13]. Further identification of hypothermia-sensitive pathological process can move ahead this perspective approach.

Glutamate is the main excitatory neurotransmitter in the mammalian central nervous system, which provides and underlies major aspects of normal brain functioning [14]. Impaired glutamate homeostasis leads to harmful neurologic consequences and is a characteristic feature of pathogenesis of neurological disorders. The extracellular glutamate levels are kept low between exocytotic events under normal physiological conditions. These levels are maintained by permanent turnover of the neurotransmitter via the plasma membrane with an input of tonic release from nerve terminals [15]. The turnover is presumably achieved by Na^+^-dependent high-affinity glutamate transporters (EAAT types 1-3), which utilize gradients of Na^+^ and K^+^ across the plasma membrane as a driving force. Neurotransmitters are accumulated and stored in synaptic vesicles, the acidic compartments of nerve terminals, that release their contents by exocytosis upon stimulation. In cerebral hypoxia, ischemia, stroke, and traumatic brain injury, the development of neurotoxicity is provoked by excesses of extracellular glutamate in the synaptic cleft released through glutamate transporter reversal [16].

Temperature influences the properties of the neuronal membrane, postpotential synaptic responses, and release of neurotransmitters (assessed by measuring excitatory postsynaptic potential), and these temperature-dependent changes in electrophysiological properties can result from effects on ion channels of the nerve cells. In fact, some calcium and voltage-gated sodium channels are regulated by temperature [7, 17–20]. Using microdialysis, Berger [10] demonstrated that moderate hypothermia (33 °C) decreased the concentration of ambient glutamate, pyruvate, lactate, and glycerol in the “tissue at risk” area of the infarct but not within the infarct core. Low body temperature appears to be associated with less release of glutamate in humans that is a marker of ischemia-induced damage of the brain [9].

Many questions are still needed to be answered regarding the use of therapeutic hypothermia, the most important of which is justification of optimal temperature regime. The aim of this study was the discrimination of the effects of hypothermia on non-pathological and pathological mechanisms of presynaptic glutamate transport using rat brain nerve terminals (synaptosomes) and assessment of potential contribution of these results to predictive, preventive and personalized medicine (PPPM). In the study: (*) we analyzed comparatively the dynamics of permanent glutamate turnover between the episodes of exocytosis measuring tonic release, uptake, and the extracellular level of glutamate under conditions of deep and profound hypothermia, and so identified hypothermia-induced changes in ischemia-unaffected nerve terminals, and (**) we assessed the velocity of pathological glutamate transporter reversal induced by membrane depolarization and the application of the protonophore carbonyl cyanide-p-trifluoromethoxyphenyl-hydrazon (FCCP) under the same conditions. The latest approach allowed identifying hypothermia-induced changes in ischemia-injured nerve terminals.

## Methods

### Ethics declaration

Wistar rats, males, body weight of 100–120 g, were kept in the institute in a quiet, temperature-controlled room at 22–23 °C using special facilities for animals, where they were provided ad libitum with water and dry food pellets. The experimental procedures were conducted according to the Helsinki Declaration “scientific requirements and research protocols” and “research ethics committees.” Experimental protocols were approved by the Animal Care and Use Committee of the Institute (protocol from 19/09-2011). Animals were sacrificed by rapid decapitation [21]. The total number of animals used in this study was 56, i.e., the assessment of l-[^14^C]glutamate uptake - 10 animals; release - 16 animals; the extracellular level - 30 animals (different parameters were measured simultaneously using one synaptosome preparation in several experiments). All experiments with rats were performed in accordance to the ARRIVE guidelines for reporting experiments involving animals [22, 23].

### Isolation of nerve terminals (synaptosomes) from rat brain

Cerebral hemispheres were quickly isolated and homogenized in ice-cold solution containing sucrose (0.32 M), HEPES-NaOH, pH 7.4 (5 mM), and EDTA (0.2 mM). One animal was used to obtain one synaptosome preparation. All procedures were performed at +4 °C. The synaptosome preparations were obtained by differential centrifugation of rat brain homogenate and then Ficoll-400 density gradient centrifugation in accordance to Cotman [24] with minor modifications [25–27]. The experiments using synaptosome preparations can be carried out during 2–4 h after isolation. The standard salt solution consisted of the following (in mM): NaCl (126); KCl (5); MgCl_2_ (2.0); NaH_2_PO_4_ (1.0); HEPES, pH 7.4 (20); d-glucose (10); the solution was oxygenated in all experiments. Ca^2+^-containing media was supplemented with CaCl_2_ (2 mM), whereas Ca^2+^-free media did not contain Ca^2+^ but was supplemented with EGTA (1 mM). Concentration of proteins was determined in accordance to Larson [28].

### Treatment of nerve terminals with hypothermia

The synaptosomal suspension obtained as described in the previous subsection was used for hypothermia experiments. Hypothermia is classified according to [29] (where deep hypothermia is considered to be from 20 °C up to 28 °C and profound from 5 °C up to 20 °C). The measurements were performed both by warming of cold synaptosomal suspension from 4 °C up to 17, 27, and 37 °C and vice versa by cooling of preliminary warmed synaptosomal suspension from 37 °C to 27 and 17 °C (see below uptake and release experiments).

### Dynamic light scattering approach

Analysis of the size of synaptosomes under hypothermia conditions was performed by dynamic light scattering using a laser correlation spectrometer “ZetaSizer-3” from Malvern Instrument, UK, equipped with He-Ne laser LGN-111, *P* = 25 mW, *λ* = 633 nm, at scattering angle 90°, and with multi-computing correlator type 7032 ce synaptosome preparations, sample volume of 1 ml, were placed in a thermostated cylindrical quartz cuvette, in which diameter is 10 mm. Synaptosome suspensions were repetitively measured for 120 s. Processing of experimental results were performed using computer software service PCS-Size mode v1.61.

### Recording of l-[^14^C]glutamate uptake by nerve terminals

Uptake of l-[^14^C]glutamate by synaptosome preparations was measured as follows. Synaptosomes (sample volume of 125 μl; 0.4 mg of protein per milliliter) were pre-incubated in the standard salt solution at 37 °C for 8 min (typical experimental approach to restore ionic gradients). Then, the suspensions were cooled to 27 and 17 °C to reach hypothermia conditions. Also, series of the experiments were performed without abovementioned preliminary incubation of synaptosomes at 37 °C. In these experiments, cold synaptosomes were warmed up to 17, 27, and 37 °C. In both approaches, uptake was started by the application of the mixture of l-glutamate (10 μM) supplemented with l-[^14^C]glutamate (420 nM, 0.1 μCi/ml), synaptosome preparations were incubated during 1 min, and then quickly sedimented at 10,000 *g* for 20 s using a microcentrifuge. l-[^14^C]glutamate uptake by synaptosomes was calculated as a lowering of radioactivity in aliquots of the supernatant (sample volume of 100 μl) and a radioactivity augmentation in the SDS-treated pellets. Amount of radioactivity was detected using ACS scintillation cocktail (volume per sample of 1.5 ml) and liquid scintillation counting [21, 30, 31]. Data were collected from several independent experiments performed in triplicate with different synaptosome preparations (*n*). Data were presented as mean ± SEM.

### Recording of l-[^14^C]glutamate release from nerve terminals

Synaptosome preparations were diluted to 2 mg of protein per milliliter and after 10 min preincubation at 37 °C were loaded with radiolabeled l-[^14^C]glutamate, 1 nmol/mg of protein, and 238 mCi/mmol in Ca^2+^-containing media at 37 °C during 10 min. Then, the synaptosomes were washed with 10 volumes of ice-cold standard salt solution; the pellets were collected and resuspended (final protein concentration of 1 mg/ml). The synaptosome preparations (sample volume of 125 μl, 0.5 mg of protein/ml) were pre-incubated at 37 °C for 8 min (typical experimental procedure for restoration of ionic gradients). After that, the synaptosome preparations were cooled to 27 and 17 °C to reach hypothermia conditions. Also, series of experiments were performed without abovementioned preliminary incubation at 37 °C. In these experiments, cold synaptosomes were warmed up to 17, 27, and 37 °C. Experiments on release of l-[^14^C]glutamate were carried out in Ca^2+^-free incubation media, where synaptosomes were incubated for different time intervals 0–30 min and then rapidly sedimented at 10,000 × *g* for 20 s in a microcentrifuge. Release was recorded in 100 μl of aliquots of the supernatant and the SDS-treated pellets using scintillation cocktail ACS (volume per sample of 1.5 ml) and liquid scintillation counting. Total l-[^14^C]glutamate amount in the synaptosomes was 200,000 ± 15,000 cpm/mg protein. For the assessment of tonic release of the neurotransmitter, the synaptosomes were incubated without stimulating agents. DL-TBOA and FCCP were added to synaptosomes at 0-time point.

### Statistical analysis

Results were presented as mean ± SEM of *n* independent experiments. Statistical differences were confirmed by two-tailed Student’s *t* test. Difference was considered significant, when *P* ≤ 0.05.

### Materials

HEPES, EGTA, EDTA, DL-TBOA, FCCP, Ficoll 400, and analytical grade salts were purchased from Sigma (USA); ACS—from Amersham (UK); radiolabelled l-[^14^C]glutamate—from Amersham (UK) and PerkinElmer (USA).

## Results

### Rationale

The majority of the experimental methods usually applied for the analysis of key characteristics of glutamatergic neurotransmission cannot be used in hypothermia-related studies because they are temperature-sensitive per se. For example, measurements of the membrane potential and synaptic vesicle acidification [32] using potential- and pH-sensitive dyes, respectively, depend not only on temperature-dependent changes in above characteristics but also on the ability of the dye to penetrate the hypothermia-affected plasma membrane etc. Therefore, such experimental data can be incorrect or need additional severe controls. The methodological approach, which allows to measure directly and adequately temperature-dependent changes in presynaptic neurotransmitter transport, involves the use of radiolabelled neurotransmitters.

The experiments on the analysis of hypothermia-induced effects were subdivided in two groups. The first one related to non-pathological processes in normal nerve terminals, that is, assessment of the uptake, tonic release, and the extracellular level of glutamate without and in the presence of glutamate transporter inhibitor DL-threo-*β*-benzyloxyaspartate (DL-TBOA) [33]. The second one was associated with pathological ischemia-related transporter-mediated glutamate release induced by depolarization of the plasma membrane of nerve terminals and dissipation of the proton gradient of synaptic vesicles. This mechanism significantly contributes to the development of excitotoxicity under hypoxia/ischemia conditions.

The experiments were conducted in the suspension of nerve terminals (synaptosomes) isolated from rat brain cerebral hemispheres. They retain major characteristics of intact nerve terminals: (*) the ability to maintain the membrane potential and the proton gradient of synaptic vesicles, (**) accomplish uptake and transporter-mediated release of glutamate, (***) exocytosis, etc. Synaptosomes are considered as one of the best systems to study presynaptic processes [34].

### Analysis of the size of nerve terminals by dynamic light scattering under conditions of deep and profound hypothermia

**Fig. 1:**
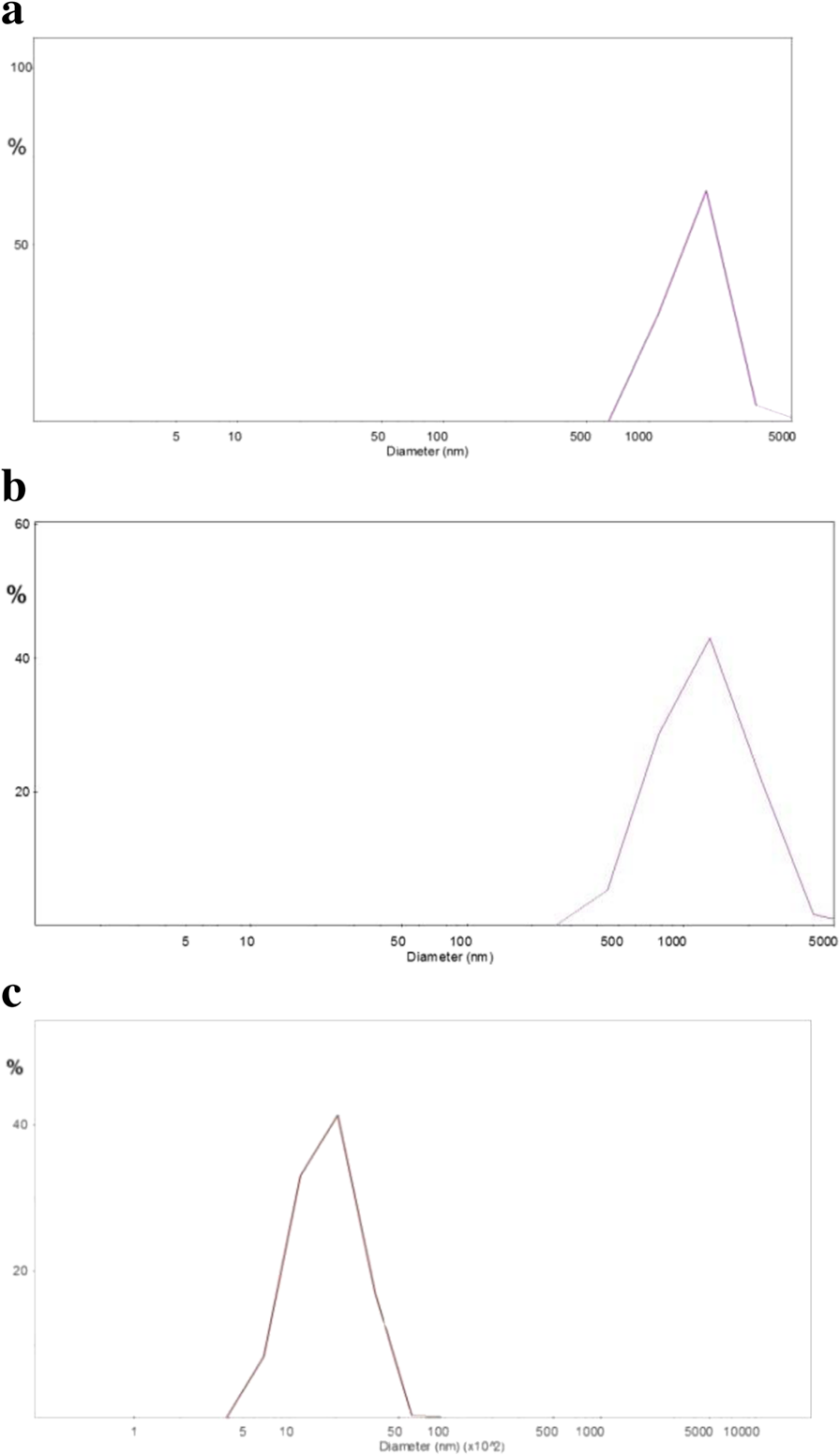
Dynamic light scattering histograms. Synaptosomal suspension (0.2 mg/ml) in a standard saline solution at 37 (**a**), 27 (**b**), and 17° C (**c**)

The average diameter of the particles in the synaptosome preparations was evaluated using dynamic light scattering in order to assess whether or not deep/profound hypothermia caused changes in the size of synaptosomes or their aggregation. As shown in Fig. 1, the size of synaptosomes (protein concentration of 0.5 mg/ml) was equal to 2.3 ± 0.3 μm in control at 37 °C, 2.4 ± 0.1 μm at 27 °C, and 2.3 ± 0.3 μm at 17 °C. Therefore, significant changes were not found in the size of synaptosomes at 27 and 17 °C in comparison with 37 °C, and so exposure of synaptosomes to above temperature conditions did not cause their aggregation at the concentrations used.

### Discrimination of hypothermia-induced changes in non-pathological mechanisms of glutamate transport in brain nerve terminals

#### Tonic release of glutamate from nerve terminals under conditions of deep and profound hypothermia

**Fig. 2:**
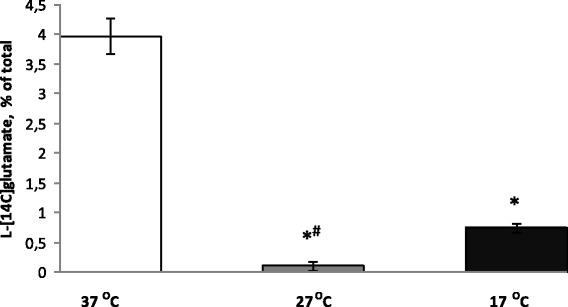
Tonic release of l-[^14^C]glutamate from nerve terminals under conditions of deep and profound hypothermia. 37 (*empty column*), at 27 (*grey column*), and 17 °C (*black column*). l-[^14^C]glutamate release was measured as described in the “Methods” section. Total synaptosomal l-[^14^C]glutamate content was equal to 200,000 ± 15,000 cpm/mg protein. Data are means ± SEM of 30 independent experiments, each performed in triplicate. Data are compared by Student’s *t* test. *, *P* ≤ 0.001 as compared to synaptosomes at 37 °C; #, as compared to synaptosomes at 17 °C

Definite level of extracellular glutamate is very important for correct synaptic transmission, and appears to be unique individual characteristic of each synapse [35]. It is clear that temperature-induced changes in the extracellular level of l-[^14^C]glutamate depend on a dynamic balance of oppositely directed processes, that is, uptake and release (see “Background” and “Discussion” sections). In the first set of the experiments, tonic glutamate release from nerve terminals, i.e. one of the abovementioned constituents determined the extracellular level of glutamate, was assessed. Analysis of tonic release of l-[^14^C]glutamate from synaptosomes revealed that it decreased in conjunction with a reduction of temperature and consisted of 3.9 ± 0.3 % of total synaptosomal label at 37 °C, 0.1 ± 0.08 % of total synaptosomal label at 27 °C (*P* ≤ 0.001, Student’s *t* test, *n* = 20), and 0.74 ± 0.08% of total synaptosomal label at 17 °C (*P* ≤ 0.001, Student’s *t* test, *n* = 20). Tonic release of l-[^14^C]glutamate from synaptosome preparations was calculated for 6 min, i.e., between 8- and 14-min time points, starting from warming of synaptosomes from +4 to 17, 27, and 37 °C, and vice versa by cooling from 37 to 27 and 17 °C. Therefore, tonic release of l-[^14^C]glutamate significantly decreased under conditions of hypothermia, and unexpectedly, this decrease was more considerable in deep than in profound hypothermia (Fig. 2).

#### Transporter-mediated glutamate uptake by nerve terminals under conditions of deep and profound hypothermia

**Fig. 3:**
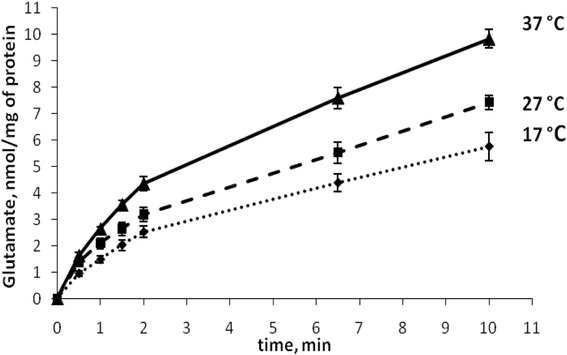
Time course of l-[^14^C]glutamate uptake by nerve terminals under conditions of deep and profound hypothermia. 37 (*solid line*), 27 (*dash line*), and 17 °C (*dotted line*). l-[^14^C]glutamate uptake was measured as described in the “Methods” section

As shown in Fig. 3, deep and profound hypothermia caused significant changes in the initial velocity of l-[^14^C]glutamate uptake by synaptosomes. The initial velocity of l-[^14^C]glutamate uptake by synaptosomes was equal to 2.63 ± 0.08 nmol min^−1^ mg^−1^ protein at 37 °C, decreased to 2.09 ± 0.2 nmol min^−1^ mg^−1^ protein at 27 °C, and further decreased to 1.48 ± 0.12 nmol min^−1^ mg^−1^ protein at 17 °C (*P* ≤ 0.05, Student’s *t* test, *n* = 6). Accumulation of l-[^14^C]glutamate by synaptosomes for 10 min consisted of 9.83 ± 0.35 nmol mg^−1^ protein at 37 °C, 7.42 ± 0.27 nmol min^−1^ protein at 27 °C, and 5.75 ± 0.52 nmol mg^−1^ protein at 17 °C (*P* ≤ 0.05, Student’s *t* test, *n* = 6). Therefore, deep and profound hypothermia significantly inhibited l-[^14^C]glutamate uptake by synaptosomes.

#### The extracellular level of glutamate in the preparations of nerve terminals under conditions of deep and profound hypothermia

**Fig. 4:**
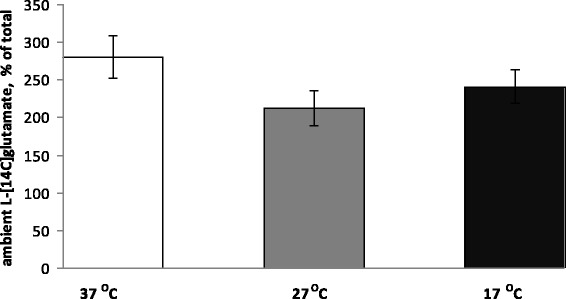
The extracellular level of l-[^14^C]glutamate in the synaptosomal suspension under conditions of deep and profound hypothermia. The level was measured at 14-min time point (see the “Methods” section) at 37 (*empty column*), 27 (*grey column*), and 17 °C (*black column*). Total synaptosomal l-[^14^C]glutamate content was equal to 200,000 ± 15,000 cpm/mg protein. Data are means ± SEM of 30 independent experiments, each performed in triplicate

**Fig. 5:**
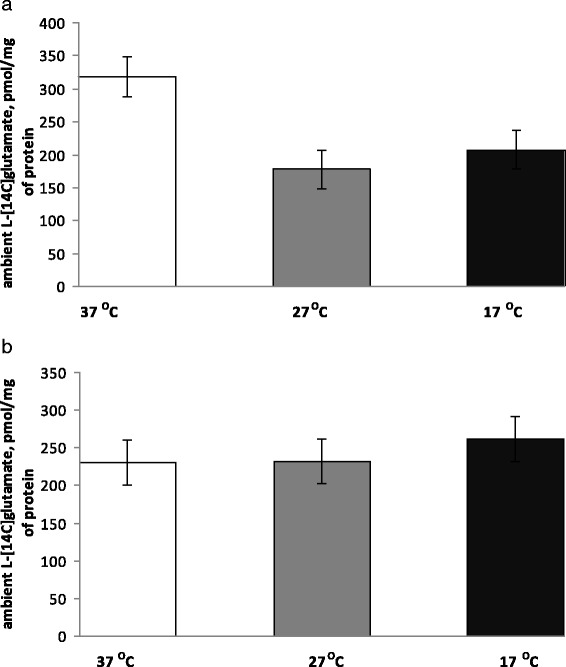
Two groups of animals with different changes in the extracellular level of l-[^14^C]glutamate in the synaptosomal suspension under conditions of deep and profound hypothermia. The experiments were subdivided in two groups with a tendency to decrease (**a**) and increase (**b**) in the value of extracellular l-[^14^C]glutamate in the synaptosomal suspension in hypothermia. The level was measured at 14-min time point (see the “Methods” section) at 37 (*empty columns*), 27 (*grey columns*), and 17 °C (*black columns*). Total synaptosomal l-[^14^C]glutamate content was equal to 200,000 ± 15,000 cpm/mg protein. Data are means ± SEM of 30 independent experiments, each performed in triplicate

As it was indicated in the previous sections, definite extracellular glutamate concentration in nerve terminals is maintained by oppositely directed processes, that is, uptake and release. The extracellular level of l-[^14^C]glutamate in the synaptosome preparations was measured at 14-min time point after warming or cooling of synaptosomes (see “Methods” section). To obtain representative experimental data, more than 30 experiments with synaptosomal preparations isolated from different animals were performed that were an order more than for usual measurements per parameter (Fig. 4). It was calculated that in a half of the experiments extracellular l-[^14^C]glutamate was decreased in hypothermia (Fig. 5a), and in a half—it was increased (Fig. 5b). Statistical analysis of the experimental data from both groups revealed the absence of significance between the control and hypothermia. This fact can be explained by the uniqueness of release/uptake balance established in each synapse, which is determined by individual synaptic characteristics such as the amount and distribution of glutamate transporters, lipid composition of the plasma membrane, and energetical status of nerve terminals. As a result, in one animal, a decrease in uptake prevails on a decrease in tonic release of l-[^14^C]glutamate and in other one vice versa. As shown in Fig. 4, the average extracellular level of l-[^14^C]glutamate was changed insignificantly as a result of a decrease in the temperature. At 14-min time point after warming/cooling of cold/warm synaptosomes, it consisted of 280 ± 28 pmol/mg of protein at 37 °C, 212 ± 23 pmol/mg of protein at 27 °C, and 241 ± 23 pmol/mg of protein at 17 °C (*n* = 30) (Fig. 4). Therefore, it was concluded that in comparison to significantly inhibited uptake and tonic release of l-[^14^C]glutamate, the extracellular l-[^14^C]glutamate level was changed insignificantly under deep/profound hypothermia. The only explanation of this fact is that tonic release decreased with comparable effectiveness with l-[^14^C]glutamate uptake, and so lowering of l-[^14^C]glutamate leakage competed with weakness of uptake.

The next set of the experiments was performed for assessment of hypothermia-induced changes in the value of the extracellular level of l-[^14^C]glutamate in the suspension of nerve terminals during blockage of glutamate transporter functioning by competitive non-transportable inhibitor of glutamate transporters DL-threo-*β*-benzyloxyaspartate (DL-TBOA) [33]. The extracellular level of l-[^14^C]glutamate in synaptosome preparations significantly decreased in deep/profound hypothermia under conditions of inhibition of glutamate uptake. No significant difference in extracellular l-[^14^C]glutamate in synaptosome preparations in the presence of glutamate transporter blocker was revealed between deep and profound hypothermia.

### Discrimination of hypothermia-induced changes in pathological ischemia-related mechanisms of glutamate transport in brain nerve terminals

#### Stimulated by depolarization of the plasma membrane transporter-mediated release of glutamate from nerve terminals under conditions of deep and profound hypothermia

**Fig. 6:**
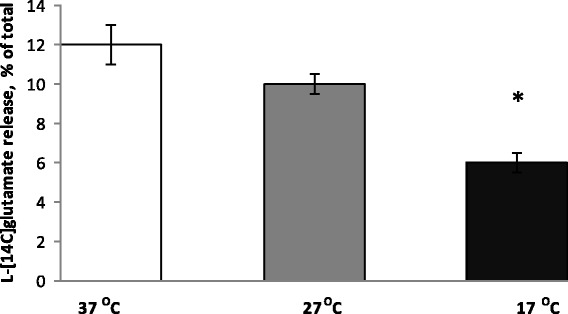
Stimulated by high KCl (35 mM) transporter-mediated release of l-[^14^C]glutamate from nerve terminals under conditions of deep and profound hypothermia. Release was measured at 37 (*empty columns*), 27 (*grey columns*), and 17 °C (*black columns*). l-[^14^C]glutamate release was measured as described in the “Methods” section, and l-[^14^C]glutamate radioactivity was determined at 6-min time point. Data are means ± SEM of five independent experiments, each performed in triplicate. *, *P* ≤ 0.05 as compared to the control

In hypoxia, ischemia, stroke, brain trauma, hypoglycemia, etc, an increase in the extracellular glutamate level causes neurotoxicity and neuronal death. Transporter-mediated glutamate release is the main mechanism that leads to an augmentation of the extracellular glutamate concentration under these pathological conditions. Depolarization of the plasma membrane of nerve terminals by high KCl in Ca^2+^-free medium causes reversal of glutamate transporters and transporter-mediated release of glutamate from the cytosol. It was shown that stimulation by high KCl release of l-[^14^C]glutamate decreased in a progressive way under conditions of deep and profound hypothermia. The value of this release measured at 6-min time point was equal to 12 ± 1% of total accumulated label at 37 °C, 10.0 ± 0.5% of total accumulated label at 27 °C, and 6.0 ± 0.5% of total accumulated label at 17 °C (*P* ≤ 0.05, Student’s *t* test, *n* = 5) (Fig. 6). Therefore, data on depolarization-evoked transporter-mediated release of l-[^14^C]glutamate from synaptosomes showed that the velocity of this release was decreased gradually from deep to profound hypothermia.

#### Protonophore-induced glutamate release from nerve terminals under conditions of deep and profound hypothermia

**Fig. 7:**
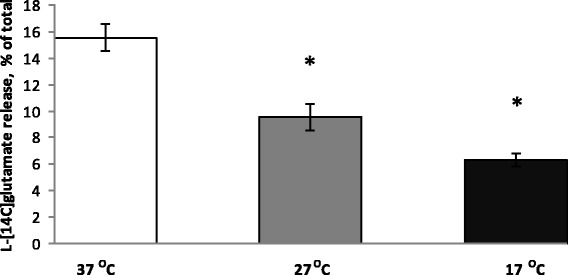
Transporter-mediated release of l-[^14^C]glutamate from nerve terminals in the presence of the protonophore FCCP (1 μM) under conditions of deep and profound hypothermia. l-[^14^C]glutamate release was measured as described in the “Methods” section, and l-[^14^C]glutamate radioactivity was determined at 6-min time point. 37 (*empty columns*), 27 (*grey columns*), and 17 °C (*black columns*). Data are means ± SEM of four independent experiments, each performed in triplicate. *, *P* ≤ 0.05 as compared to control

The protonophore FCCP is used in the experiments due to its ability to dissipate the proton gradient of synaptic vesicles and inhibit glutamate uptake [27]. These conditions favor an augmentation of transporter-mediated l-[^14^C]glutamate release from synaptosomes. FCCP-evoked l-[^14^C]glutamate release from synaptosomes was significantly inhibited by the transporter inhibitor DL-TBOA, so this release was mediated by glutamate transporters. In this set of the experiments, the effect of deep and profound hypothermia on l-[^14^C]glutamate release from synaptosomes during application of FCCP was analyzed. As shown in Fig. 7, FCCP-evoked release of l-[^14^C]glutamate for 6 min was equal to 15.5 ± 1.0% of total accumulated label at 37 °C, 9.5 ± 1.0% of total accumulated label at 27 °C (*P* ≤ 0.05, Student’s *t* test, *n* = 4), and 6.3 ± 0.5% of total accumulated label at 17 °C (*P* ≤ 0.05, Student’s *t* test, *n* = 4).

## Discussion

Glutamate-related mechanism of neuroprotective action of hypothermia is still debated despite a lot of experimental data [7, 10, 18, 19, 36]. The explanation of the possible neuroprotective effect of lowering body temperature in acute stroke remains largely speculative [9]. However, most studies point toward lowering down of the neurodegenerative processes in the penumbra [18, 19, 37, 38]. Also, there are no clear standards of the parameters in therapeutic hypothermia [13], and the optimal temperature for hypothermic circulatory arrest during arch surgery remains unclear [1].

In this study, double discrimination of the effects of deep and profound hypothermia (the first type of discrimination) on non-pathological and pathological mechanisms (the second type of discrimination) of presynaptic l-[^14^C]glutamate transport was performed using rat brain nerve terminals. According to the recent hypothesis forwarded by the author of this study, a definite and non-negligible concentration of extracellular glutamate between the episodes of exocytotic release is established mainly by permanent transporter-mediated glutamate turnover across the plasma membrane of nerve terminals with a contribution of non-transporter tonic glutamate release by spontaneous exocytosis, diffusion, cystine-glutamate exchanger, and leakage through anion channels [15]. Nevertheless, glutamate transporter reversal remains the major mechanism of glutamate release under energy deprivation conditions, in hypoxia, hypoglycemia, brain trauma, stroke, underlying augmentation of the extracellular glutamate concentration, and development of excitotoxicity. Dynamics of l-[^14^C]glutamate turnover (between episodes of exocytosis) and its constitutive mechanisms, that is, high-affinity Na^+^-dependent uptake accompanied with non-pathological transporter-mediated release of l-[^14^C]glutamate, and also tonic l-[^14^C]glutamate release, were analyzed separately under conditions of deep and profound hypothermia. A balance of hyperthermia-induced changes in above constituents determines definite extracellular level of l-[^14^C]glutamate in nerve terminals under experimental conditions. It was shown that transporter-mediated l-[^14^C]glutamate uptake was decreased in progressive manner from deep to profound hypothermia (Fig. 3). Theoretical calculations predict that non-pathological glutamate transporter reversal (as a component of permanent transporter-mediated turnover) should also decrease under hypothermia. Unfortunately, this component cannot be measured directly by any methodological approaches.

Tonic release of l-[^14^C]glutamate for 6 min decreased more considerably (almost completely absent) under conditions of deep hypothermia (Fig. 2, the second column) in comparison with profound one (Fig. 2, the third column). It was clearly demonstrated that no gradual decrease in tonic l-[^14^C]glutamate release between deep and profound hypothermia was registered. Summarizing, uptake of l-[^14^C]glutamate decreased progressively (Fig. 3), whereas tonic release decreased almost similarly in deep to profound hypothermia by more than 80% (Fig. 2).

Theoretical calculations predict that if transporter-mediated l-[^14^C]glutamate uptake is more sensitive to deep and profound hypothermia than the sum of non-pathological transporter-mediated and non-transporter tonic release of l-[^14^C]glutamate, the extracellular level of l-[^14^C]glutamate should increase in the preparation of nerve terminals and vice versa if release is more sensitive—this level should decrease. Taking ten times more animals than for ordinary series of the experiments, it was shown that the ability to maintain the balance glutamate_in_/glutamate_out_ in hypothermia, and so extracellular level of l-[^14^C]glutamate was individual for each animal. It is so because in 50% of experiments, the extracellular l-[^14^C]glutamate level was decreased under hypothermia (Fig. 5a) and in 50%—it was increased (Fig. 5b). As a result, the statistic calculation showed the absence of significance in hypothermia-dependent decrease in the extracellular level of l-[^14^C]glutamate in the preparation of nerve terminals (Fig. 4).

Absence of significant changes in extracellular l-[^14^C]glutamate in the preparation of nerve terminals despite of reduced l-[^14^C]glutamate uptake during lowering of temperature is valuable per se. It is clear from these hypothermia experiments that release components (tonic and non-pathological transporter reversal) have significant contribution to the establishment and maintenance of proper extracellular glutamate concentration. Variability of hypothermia-induced changes in extracellular l-[^14^C]glutamate shown in this study favors our recent suggestion regarding individuality and uniqueness of the extracellular level of the neurotransmitters for each synapse [35].

Therefore, the effect of therapeutic hypothermia on the extracellular level of glutamate in nerve terminals unaffected by hypoxia/ischemia cannot be predicted a priori. Taking into account the above fact, it is clear that the changes in the extracellular level of glutamate during hypothermia are unique for each patient, and so the individual approach of the evaluation of hypothermia parameters should be developed.

Our data coincides with the results of Boris–Moller and Wieloch [39], who found an attenuation of glutamate levels only in the striatum, whereas cortical ones, although were lower at the baseline, but were not attenuated by hypothermia. However, the experimental data is not in accord with microdialysis measurements that demonstrated that glutamate dialysate concentrations in non-infarcted brain tissue decreased from 3.6 to 2.6 mol/L in mild hypothermia [10]. Only a tendency to decrease in synaptosomal extracellular l-[^14^C]glutamate was found in our experiments.

This study also focused on the analysis of the velocity of pathological glutamate transporter reversal induced by membrane depolarization and FCCP that reflected hypothermia-induced changes in ischemia-injured nerve terminals. Development of neurotoxicity in stroke, cerebral hypoxia, and ischemia, cardio surgery with circulation arrest, and traumatic brain injury is provoked by excessive extracellular glutamate mainly originated from glutamate transporter reversal. Glutamate transporter reversal was stimulated by depolarization of the plasma membrane by 35 mM KCl (Fig. 6) and by dissipation of the proton gradient of synaptic vesicles with the protonophore FCCP (Fig. 7), and a gradual decrease from deep to profound hypothermia in the velocity of transporter-mediated l-[^14^C]glutamate release from synaptosomes was shown. This fact indicates a progressive neuroprotective effect increased from deep to profound hypothermia in affected by hypoxia/ischemia zones, e.g., in core and penumbra zones of the insult. This experimental data coincides with the results of [40], where the authors analyzing the dialysate concentrations showed that the rate of an ischemia-induced increase in glutamate concentration was inhibited by low temperature, and was equal to 58.4 ± 31.8 μmol/L at 38 °C to 15.9 ± 8.4 μmol/L at 25 °C. Also, our results agree with those of Berger [10], where the authors demonstrated using microdialysis measurements that glutamate dialysate concentrations in peri-infarct tissue were significantly influenced by hypothermia (extracellular glutamate, 4.8 versus 12.6 mol/L). In this context, we partially agree with the suggestion that hypothermia exerts a neuroprotective effect by extracellular glutamate attenuation in penumbral regions and possibly within the infarct core. The present study brings new insight to the above statement, namely, it is true, when the nerve cells are still able to release glutamate by transporter reversal (Figs. 6 and 7) and also by non-transporter mechanisms (Fig. 2). In considerably injured nerve cells, the above mechanisms do not work because of thermodynamic reasons, i.e., reduction of the glutamate_in_/glutamate_out_ gradient across the plasma membrane. In this context, selection of optimal individual temperature regime for patients in dependence of the size of irreversibly injured zone and extent of penumbra one is of value.

Taking into account abovementioned facts, it is suggested that the strategy of successful therapeutic hypothermia from one side is a reduction of its possible harmful effects on hypoxia/ischemia-unaffected nerve cells by stabilization of the extracellular glutamate concentration through the balancing release/uptake constituents. It is rational to make hypothermia-induced decrease in glutamate transporter reversal more significant, thereby enhancing its direct neuroprotective effect. Above recommendations can be realized by combination of unspecific effects of hypothermia with other unspecific and specific neuroprotectants.

In medical practice, there are no specific agents for modulation of glutamate transporter activity, in contrast to selective inhibitors of GAT1 and GAT3 GABA transporters, tiagabine, and β-alanine, respectively. In this context, unspecific modulation of transporter activity is the only available approach to decrease significantly pathological transporter-mediated glutamate release from nerve terminals. Unspecific neuroprotection can be performed by hypothermia and also by temporal cholesterol deficiency. Recently, we have shown that a decrease in the level of membrane cholesterol in nerve terminals reduced pathological transporter-mediated glutamate release. These data can explain the neuroprotective effect followed by the administration of statins in excitotoxicity, stroke, cerebral hypoxia and ischemia, seizures, oxidative damage, and traumatic brain injury. However, we underline that except abovementioned pathologies, the normal levels of membrane cholesterol are extremely important for correct synaptic transmission and a decrease in membrane cholesterol content of nerve terminals may cause neurotoxic consequences because of weak glutamate uptake and the enlargement of the extracellular glutamate concentration [32, 41, 42]. Also, nanoparticles can be perspective for modulation of glutamate transport and visualization of key processes in nerve terminals [43].

### Limitations of the study

The main limitation of this study is associated with the fact that data was obtained from in vitro experiments. Nevertheless, the existence of pathological glutamate transporter reversal and importance of the extracellular glutamate level is commonly accepted [10, 14, 16].

### Recommendations for PPPM

The results of this study are of value for PPPM, a medicine that focuses on the combined diagnosis, prevention, and treatment of disease in individual patients. Discrimination of hypothermia-induced changes in non-pathological and pathological glutamate transport in nerve terminals, proven diversity of above hypothermia-induced effects, and individuality of changes in extracellular glutamate underscores usefulness of the result implementation in PPPM. It is suggested from our study that the neurological complications in cardiac surgery can be associated with an increase extracellular glutamate level. In “healthy” nerve terminals, hypothermia-induced changes in the extracellular glutamate level cannot be predicted because hypothermia sensitivity of uptake vs. tonic release cannot be assessed by known approaches. However, blood platelets are able to accomplish glutamate uptake by neuronal-like high-affinity Na^+^-dependent glutamate transporters EAAT 1-3 of the plasma membrane [35]. This feature of platelets may be used for characterization of hypothermia sensitivity of glutamate transport in brain nerve terminals. Similarities of platelets with nerve terminals underlie possible usage of platelets as a model of glutamate transport in the presynapse and an analytic, diagnostic, and prognostic biomarker for the analysis of the alterations in the functioning of glutamate transporters in the brain [35]. Interrelation between changes in glutamate transport and metabolism in the brain and platelets was shown [44–47], and so the possible role of platelets as a diagnostic and prognostic marker in various neurodegenerative diseases was suggested [48]. The probable physiological role of platelets in extracellular glutamate homeostasis in the central nervous system is realized by removing of excessive extracellular glutamate [49–52]. The importance of proper glutamate concentration in the blood was demonstrated in [53].

Taking into account our data on significant gradual decrease in pathological glutamate transporter reversal, it is clear that the more size of penumbra zone, the less temperature in therapeutic hypothermia should be applied. Preliminary diagnostics and neuromonitoring should be an essential part of hypothermia therapy helping physicians to choose optimal temperature regime. Increased plasma glutamate in stroke patients was registered [48]. In this context, non-neuronal glutamate can serve as a diagnostic, prognostic, and predictive biomarker, and glutamate biosensor [31] can be applied in medical practice. In contrast, platelets cannot be included in neuromonitoring of pathological glutamate transporter reversal, because we recently showed that this manner of glutamate release in platelets is rather ambiguous [35] and glutamate is released from platelets exclusively by means of exocytosis.

**Fig. 8:**
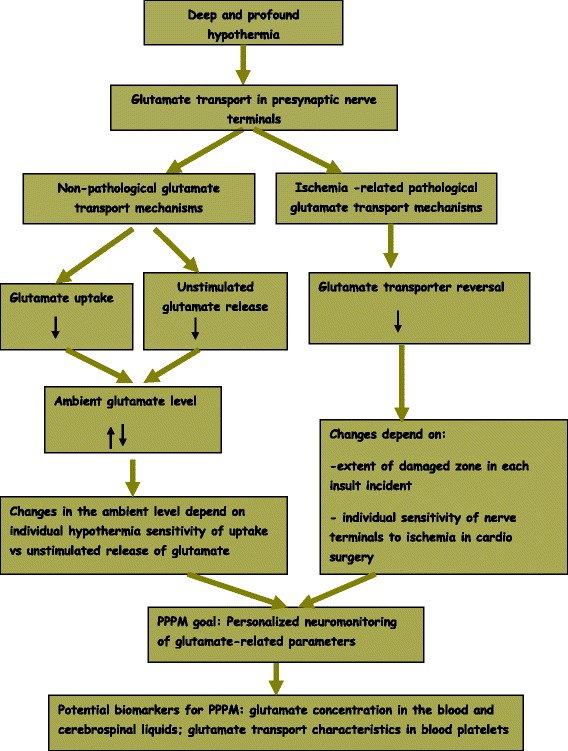
Roadmap of the study

Therefore, the PPPM goal for the future is to implement comprehensive neuromonitoring of glutamate-related parameters in the blood plasma, platelets, and cerebrospinal liquids that helps to select optimal individual temperature regime for patients with prescribed therapeutic hypothermia (Fig. 8). Validation of proposed biomarkers, i.e., glutamate concentration in the blood and cerebrospinal liquids and glutamate transport characteristics of platelets, is necessary before their incorporation into standard clinical decision-making algorithms. Personalized approach will certainly play the crucial role in the more effective and better tailored hypothermia treatment in cardiac surgery of the aortic arch and in ischemic stroke.

In perspective, we plan to develop the panels of glutamate-related biomarkers for neuromonitoring of the patients reflected individual hypothermia-induced effects in nerve terminals that will serve for the outcome prediction methodology. This plan bases on our recent results that the extracellular glutamate level is unique for each synapse [35], a possibility to analyze kinetics of glutamate uptake by nerve terminals using glutamate biosensor [31], and characteristic features of glutamate transport in blood platelets [35]. These data can be processed in appropriate way and be a base for development of new computational and mathematical tools to predict hypothermia effects.

## Conclusions

Summarizing, dual discrimination of the effects of deep and profound hypothermia on non-pathological and pathological mechanisms of presynaptic glutamate transport was performed using rat brain nerve terminals. Hypothermia-induced changes in extracellular l-[^14^C]glutamate in unaffected ischemia nerve terminals are not evident. It is so because the differently directed constituents determined physiological extracellular level, that is, glutamate uptake and tonic release both were decreased in deep and profound hypothermia.

Therefore, in unaffected nerve terminals, the direction of changes in extracellular glutamate in deep and profound hypothermia is unpredictable and depends on individual synaptic characteristics. While in affected ones (e.g., in brain regions suffering from a reduction of blood circulation during cardio surgery and core and penumbra zones of the insult), pathological transporter-mediated glutamate release decreased with progressive significance from deep to profound hypothermia, thereby underlying its unspecific potent neuroprotective effect in stroke, hypoxia and ischemia, cardiac surgery with application of circulation arrest, and traumatic brain injury, where excitotoxicity progress is associated with an increase in glutamate uptake reversal. Our data revealed the necessity of development and advance of special parameters in PPPM for individual neuromonitoring in hypothermia.

## Abbreviations

ACS: Aqueous counting scintillant
DL-TBOA: DL-threo-*β*-benzyloxyaspartate
EDTA: Ethylenediaminetetraacetic acid
EGTA: Ethylene glycol tetraacetic acid
FCCP: Carbonyl cyanide-p-trifluoromethoxyphenyl-hydrazon
GABA: γ-aminobutyric acid
GAT1 and GAT3: γ-aminobutyric acid transporter subtypes
HEPES: 4-(2-hydroxyethyl)-1-piperazineethanesulfonic acid
